# LAMP-2 is required for incorporating syntaxin-17 into autophagosomes and for their fusion with lysosomes

**DOI:** 10.1242/bio.018648

**Published:** 2016-09-14

**Authors:** Virginie Hubert, Andrea Peschel, Brigitte Langer, Marion Gröger, Andrew Rees, Renate Kain

**Affiliations:** 1 Clinical Institute of Pathology, Medical University of Vienna, Vienna1090, Austria; 2 Core Facilities, Medical University of Vienna, Vienna 1090, Austria

**Keywords:** LAMP-2, STX17, Lysosome, Autophagosome, VAMP8, Macroautophagy, LC3

## Abstract

Autophagy is an evolutionarily conserved process used for removing surplus and damaged proteins and organelles from the cytoplasm. The unwanted material is incorporated into autophagosomes that eventually fuse with lysosomes, leading to the degradation of their cargo. The fusion event is mediated by the interaction between the Qa-SNARE syntaxin-17 (STX17) on autophagosomes and the R-SNARE VAMP8 on lysosomes. Cells deficient in lysosome membrane-associated protein-2 (LAMP-2) have increased numbers of autophagosomes but the underlying mechanism is poorly understood. By transfecting LAMP-2-deficient and LAMP-1/2­-double-deficient mouse embryonic fibroblasts (MEFs) with a tandem fluorescent-tagged LC3 we observed a failure of fusion between the autophagosomes and the lysosomes that could be rescued by complementation with LAMP-2A. Although we observed no change in expression and localization of VAMP8, its interacting partner STX17 was absent from autophagosomes of LAMP-2-deficient cells. Thus, LAMP-2 is essential for STX17 expression by the autophagosomes and this absence is sufficient to explain their failure to fuse with lysosomes. The results have clear implications for situations associated with a reduction of LAMP-2 expression.

## Abstract (precis)

**Summary:** LAMP-2 is required for autophagosome-lysosome fusion. Its absence does not affect the lysosomal SNARE VAMP8 while its interacting partner STX17 is absent from the autophagosomes providing a molecular explanation for this fusion failure.

## INTRODUCTION

Macroautophagy (hereafter autophagy) is characterized by unique double-membrane vacuoles that transfer surplus and damaged proteins and organelles into lysosomes for degradation. This maintains the quality of the cytoplasm and is critical for cellular homeostasis and resistance to stress. Autophagy is essential for tissue remodeling during embryogenesis and for innate and adaptive immune responses. The efficiency of autophagy declines with age and this contributes to diseases found in the elderly, including degenerative neurological disorders, autoimmune and inflammatory diseases, and tumors (Mizushima et al., 2008).

In mammalian cells, autophagy is mainly regulated by the level of amino acids and insulin that converge to mTOR, the main regulator of nutrient signaling (Mizushima, 2007). This process is initiated at the endoplasmic reticulum (ER)-mitochondria contact sites with the development of open-ended cisterna, phagophores, which incorporate cytoplasmic components, including organelles, by engulfment (Hamasaki et al., 2013) and cargo-specific loading (Deretic et al., 2015). Phagophore initiation is regulated by three multiprotein complexes: the ULK1 complex comprising ULK1, autophagy-related protein 13 (Atg13), Atg101 and FIP200; the Vps 34 complex that includes Beclin1, Atg14L and Vps34; and the Atg16/Atg5/Atg12 complex. The final stage of phagophore development involves Atg3 and the incorporation of LC3 (Atg8). LC3 occurs in two forms; an unconjugated cytoplasmic form, LC3-I, and a conjugated (lipidated) form, LC3-II, that integrates into the phagophore membrane (Nakatogawa et al., 2007). LC3 persists after membrane closure and leads to the formation of the autophagosomes (Mizushima et al., 2008), whereas other Atg proteins and early phagophore components dissociate from mature autophagosomes (Krämer, 2013). Consequently, LC3-I and LC3-II in cell lysates have been used to monitor autophagy (Kimura et al., 2007). Eventually autophagosomes fuse with lysosomes to become autolysosomes.

Fusion of autophagosomes with lysosomes is essential for autophagic flux and the mechanisms responsible are beginning to be understood (Mizushima, 2014). They involve the interactions between numerous proteins, including some specific for autophagosome/lysosome fusion and others involved in vesicular fusion more generally. Selected vesicles and organelles are brought together both by diffusion and motor-mediated transport and then tethered to each other by specific molecular interactions. This facilitates the fusion event that occurs when a v-SNARE (N-ethylmaleimide-sensitive factor attachment protein receptor) in the membrane of one vesicle engages a t-SNARE to form a bundle of four parallel core SNARE domains that approximates the two bilayers and precipitates fusion (Whyte and Munro, 2002). Tethering autophagosomes to lysosomes critically involves the Rab7 effector ORP1L and RILP, which recruits the dynein-dynactin motor and interacts with the homotypic fusion and protein sorting (HOPS) complex that consists of six subunits of vacuole sorting proteins (VPS) (Balderhaar and Ungermann, 2013; Hyttinen et al., 2013; Wijdeven et al., 2016). This interaction is tightly regulated by the cholesterol sensor ORP1L that, under low cholesterol conditions, prevents the recruitment of HOPS to Rab7-RIPL and therefore tethering of the membrane (Wijdeven et al., 2016). The HOPS complex will then tether lysosomes to autophagosomes by cross-linking Rab7 on lysosomes to syntaxin 17 (STX17) on autophagosomes (Jiang et al., 2014) in a process augmented by Atg14 (Balderhaar and Ungermann, 2013; Diao et al., 2015). STX17 is also the Qa-SNARE essential for autophagosome-lysosome fusion. After incorporation into the autophagosome membrane, STX17 recruits the Qbc-SNARE synaptosomal-associated protein 29 (SNAP-29) and the complex is then bound by oligomeric Atg14, which primes its interaction with the R-SNARE VAMP8 on the lysosomes and induces fusion (Fig. S1) (Diao et al., 2015; Itakura et al., 2012). Various other proteins have been implicated in autophagosome/lysosome fusion but their contribution is unclear (Mizushima, 2014). These include lysosome membrane protein-2 (LAMP-2), a protein with a defined role in phagolysosome biogenesis as demonstrated by a failure of opsonized latex beads to stain with a lysotracker (Huynh et al., 2007) in fibroblasts doubly deficient for LAMP-1 and LAMP-2. A specific role of LAMP-2 in autophagosome fusion has also been suggested but decisive proof is lacking (González-Polo et al., 2005).

LAMP-2 is a heavily glycosylated type-1 membrane protein with three splice variants, LAMP-2A, LAMP-2B and LAMP-2C (Carlsson et al., 1988; Eskelinen et al., 2005). The three isoforms consist of a common heavily glycosylated extracellular domain linked to a single membrane spanning domain and a short cytoplasmic tail that includes a lysosomal/endosomal targeting signature and differs between the three isoforms (Carlsson and Fukuda, 1989; Gough and Fambrough, 1997). Over the past decade LAMP-2 has been recognized to make an important contribution to an increasing number of cellular processes. Some functions appear to be shared by all isoforms – for example antigen presentation, (Zhou et al., 2005) cholesterol trafficking, (Schneede et al., 2011) lysosome biogenesis (Eskelinen et al., 2002) and phagocytosis (Huynh et al., 2007) – whilst others are isoform-specific. Thus, the essential role of LAMP-2 in chaperone-mediated autophagy (CMA) is unique to LAMP-2A (Cuervo and Dice, 1996); only LAMP-2B contributes to retention of TAPL in the lysosomal membrane (Demirel et al., 2012); and RN/DNautophagy is mediated exclusively by LAMP-2C (Fujiwara et al., 2013a,b). Human and murine cells deficient in LAMP-2 have increased numbers of autophagosomes, (Endo et al., 2015; Tanaka et al., 2000) but although previous methods such electron microscopy allow the identification of autophagosomes and autolysosomes, they could not quantify autophagic flux precluding analysis of fusion.

Transfection of cells with a LC3 construct fused to an mRFP and EGFP (tfLC3) tandem fluorescent tag identifies autophagosomes and autolysosomes and clearly distinguishes between them (Kimura et al., 2007). We have used this construct to establish that LAMP-2-deficient mouse fibroblasts have a major defect in the degradation of autophagosomal content within the lysosome that is reversed by complementation with LAMP-2A but not LAMP-2B. Remarkably, LAMP-2 deficiency did not affect the expression of the lysosomal R-SNARE VAMP8 but instead reduced the abundance of autophagosomal STX17 to near undetectable levels, and altered recruitment of the accessory proteins required for tethering and fusion. These results identify a novel function for LAMP-2 and have obvious implications for understanding the mechanisms of autophagosomal maturation and degradation and the consequences of LAMP-2 deficiency.

## RESULTS

### LAMP-2 deficiency inhibits macroautophagy

**Fig. 1.**
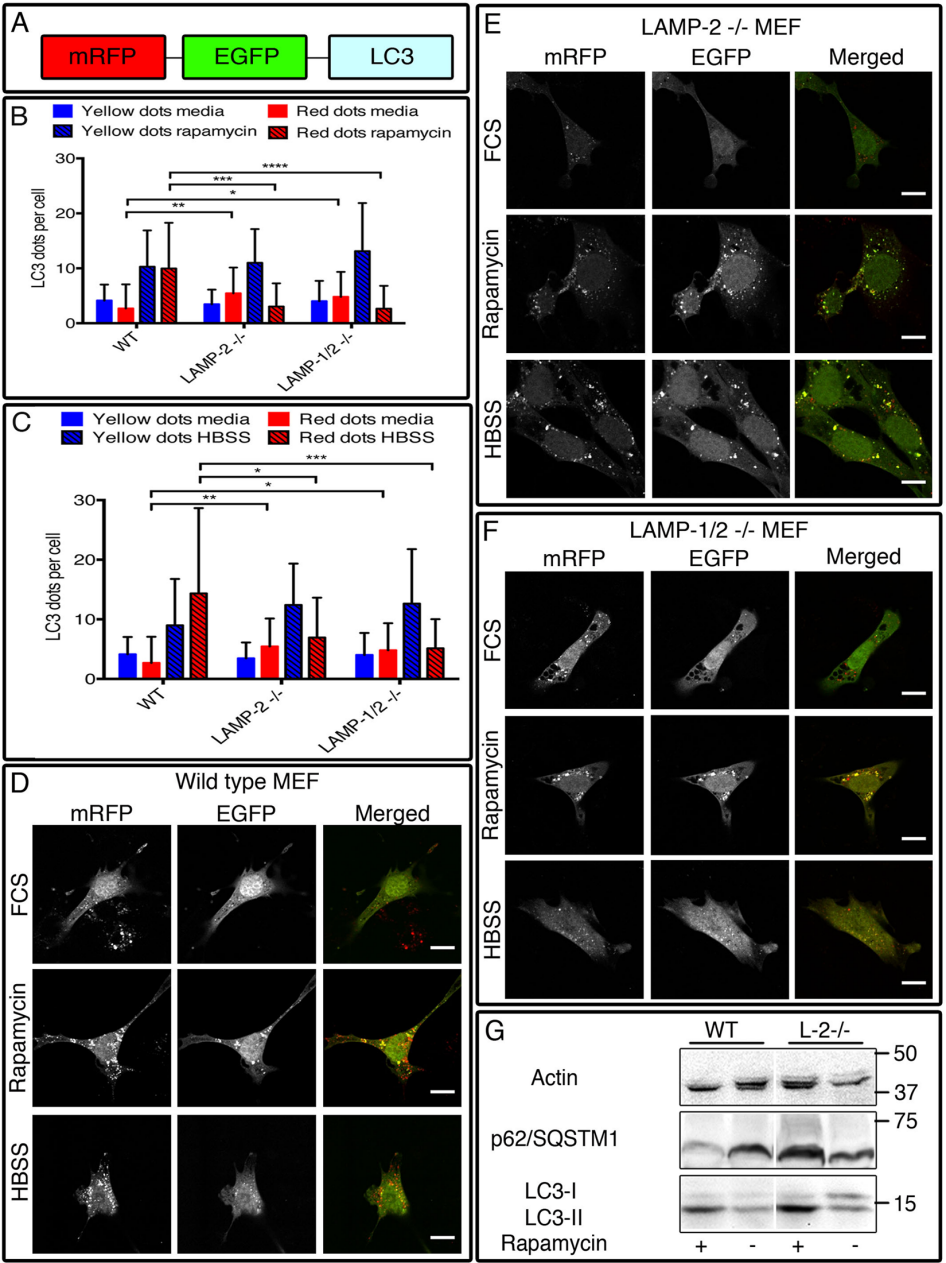
**The autophagic flux is impaired in LAMP-2-single- and LAMP-1/2-double-deficient cells after autophagy induction.** Cells were transfected with the tandem-fluorescent tagged mRFP–GFP–LC3 construct (A) and cultured in media (control), rapamycin (50 µm) (B) or HBSS (C) for 6 h. In wild-type MEFs, autophagy induction was characterized by an increased number of autophagosomes and autolysosomes (D) while in LAMP-2-single-deficient (E) and LAMP-1/2-double-deficient (F) cells the number of autolysosomes remained unchanged after treatment with rapamycin or HBSS. (G) Western blot analysis of total cell extract reveals an absence of degradation of p62 after rapamycin treatment confirming the blockage of the autophagic flux in LAMP-2-negative cells. Scale bars=10 µm. Data are expressed as mean±s.d. of three independent experiments. *P<0.05; **P<0.01; ***P<0.001; ****P<0.0001 (Mann–Whitney test).

We first established the conditions for the induction of autophagy in a well-characterized set of SV40 transformed mouse embryonic fibroblast (MEF) cell lines derived from mice singly deficient in LAMP-2, doubly deficient in LAMP-1 and LAMP-2, and wild-type littermates, and analyzed the contribution of LAMP-2 to autophagosome-lysosome fusion (Eskelinen et al., 2004). All three cell lines expressed the Simian virus 40 (SV40) large T antigen (Fig. S2A) and LAMP-1 and LAMP-2 were undetectable by immunofluorescence and western blotting in the appropriate deficient cells line (Fig. S2B,C). The MEF cell lines were then transiently transfected with tandem-tagged LC3 (tfLC3/mRFP–EGFP–LC3) (Fig. 1A) and autophagosomes that had incorporated tfLC3 were identified by orange fluorescence (co-localisation of mRFP and EGFP) that distinguished them from autolysosomes that fluoresce red (mRFP) because of quenching and digestion of the EGFP when exposed to lysosomal acidity and enzymes (Fig. S2D) (Kimura et al., 2007). This enabled us to assess the formation and the maturation of newly synthesized autophagosomes.

In wild-type MEFs, induction of autophagy after 6 h incubation with rapamycin (Fig. 1B) or Hank's balanced salt solution (HBSS) (Fig. 1C) caused parallel increases in autophagosomes and autolysosomes: autophagosomes – 4.15±2.89 to 10.29±6.61 (mean±s.d.) (*P*<0.0001) and 9.00±7.78 (*P*<0.01) puncta/cell pre- and post-rapamycin and HBSS respectively; autolysosomes – 2.70±4.37 to 9.96±8.31 (*P*<0.0001) and 14.35±14.30 puncta/cell (*P*<0.0001) pre- and post-rapamycin and HBSS (Fig. 1D). Rapamycin and HBSS caused the expected increase in autophagosomes in LAMP-2-deficient MEFs but without change in the number of autolysosomes (autophagosomes – 3.47±2.65 to 11.00±6.13 and 3.47±2.65 to 12.41±6.95 pre- and post-rapamycin and HBSS respectively; and autolysosomes – 5.47±4.67 to 3.05±4.21 and 6.95±6.68) (Fig. 1E). The results with LAMP-1/2-double-deficient MEFs were identical (Fig. 1F). Incubation with rapamycin or HBSS for 4 h produced similar results (Fig. S3A,B), thus confirming the failure to generate autolysomes in LAMP-2-deficient MEFs and implying a reduction in autophagic flux.

We confirmed the influence of LAMP-2 on autophagic flux using western blot to examine endogenous concentrations of LC3-I and LC3-II and p62/sequestosome 1 (p62/SQSTM1), a targeting molecule incorporated into autophagosomes and degraded in autolysosomes. Under resting conditions, LC3-I and LC3-II were more abundant in the lysates from LAMP-2-deficient MEFs than wild-type cells. Incubation with rapamycin slightly increased the level of LC3-II in wild-type cells and the effect was exaggerated in LAMP-2-deficient cells, consistent with the failure to degrade LC3 II (Fig. 1G). Similarly, concentrations of p62/SQSTM1 decreased in wild-type MEFs after rapamycin treatment but remained unchanged in LAMP-2-deficient cells, indicating the failure to increase autophagic flux. Collectively, the data establish that LAMP-2-deficient cells have impaired autophagic flux characterized by a selective reduction in autolysosomes. This locates the defect at a late stage of the autophagic process.

### Reconstitution with LAMP-2A restores the wild-type phenotype

**Fig. 2.**
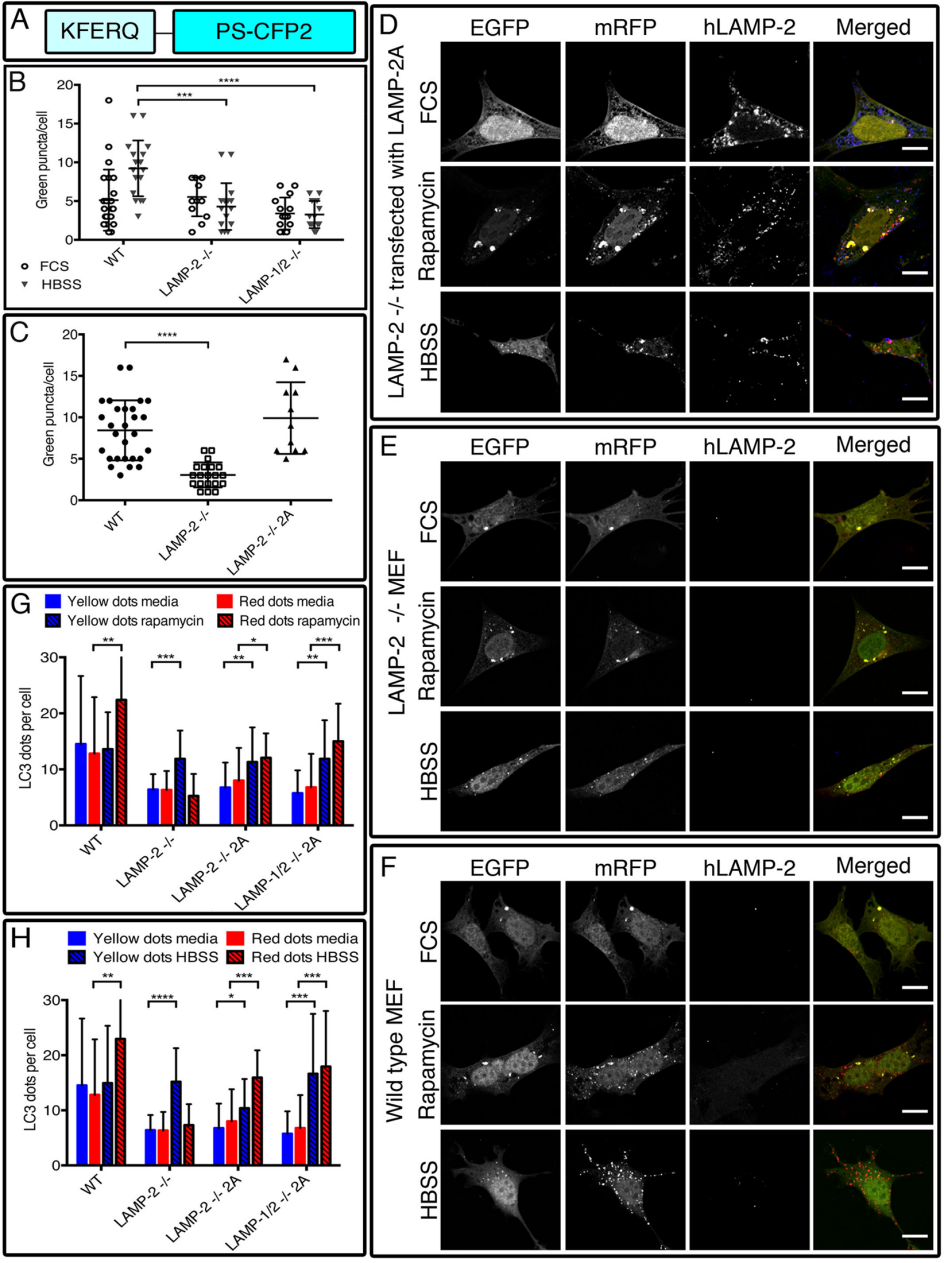
**CMA and autophagic flux are restored by LAMP-2A.** CMA, monitored with the photoswitchable reporter pKFERQ-PS-CFP2 (A) could be efficiently induced in wild-type cells but not in LAMP-2-single- and LAMP-1/2­-double-deficient MEFs indicating their inability to conduct CMA (B). This process was efficiently restored by transfection with LAMP-2A (C). Similarly, reconstitution of LAMP-2-deficient cells with LAMP-2A (D) restored the increase of autolysosomes after induction of autophagy. Results were compared to LAMP-2-deficient (E) and wild-type MEFs (F) and reveal a restoration of the autophagic flux after treatment with both rapamycin (G) or HBSS (H). Scale bars=10 µm. Data are expressed as mean±s.d. of three independent experiments. *P<0.05; **P<0.01; ***P<0.001; ****P<0.0001 (Mann–Whitney test).

Next we used human LAMP-2 cDNA constructs to establish that absence of LAMP-2 was responsible for the defect in autophagosome fusion and that the human protein was able to rescue endogenous mouse LAMP-2 deficiency. To confirm the functional efficacy of the LAMP-2 constructs, cell lines were transiently transfected with a photoswitchable CMA reporter construct, pKFERQ-PS-CFP2 (Koga et al., 2011) (Fig. 2A) which, after photoconversion, identifies lysosomes that have imported the reporter protein by LAMP-2A-dependent CMA as green fluorescent puncta. There were few green fluorescent puncta in wild-type MEFs incubated in complete medium, indicating a low basal level of CMA, but these increased greatly after 24 h incubation in HBSS (Fig. 2B; Fig. S4A). By contrast, HBSS failed to increase the number of green puncta in either LAMP-2-deficient (Fig. 2B; Fig. S4B) or LAMP-1/2-double-deficient MEFs (Fig. 2B; Fig. S4C), demonstrating their inability perform CMA (Table S1). The efficacy of the LAMP-2A construct was then evaluated by its ability to restore CMA in the deficient cell lines. This was done by transiently co-transfecting the cell lines with the photoswitchable CMA reporter construct and LAMP-2A; the effectiveness of transfection was confirmed by immunofluorescence (Fig. S4D). Complementing with LAMP-2A completely restored the defect in CMA (Fig. 2C; Table S2) and therefore confirms the functional efficacy of the construct. This enabled us to test whether restoring LAMP-2 expression was equally effective at overcoming the defect in autophagy.

LAMP-2-deficient MEFs were co-transfected with tfLC3 and either LAMP-2A (Fig. 2D) or LAMP-2B (Fig. S5A) and expression of the transgene was confirmed by indirect immunofluorescence. Using an antibody specific for hLAMP-2, the results were compared to those obtained with uncomplemented LAMP-2-deficient MEFs (Fig. 2E) and wild-type MEFs (Fig. 2F). As with CMA, complementation with LAMP-2A rectified the defect in autophagy as demonstrated by the concomitant increase in autophagosomes and autolysosomes after rapamycin treatment, both in LAMP-2-single- and LAMP-1/2-double-deficient MEFs (Fig. 2G). Autophagic flux induced by HBSS was similarly restored (Fig. 2H). By contrast, reconstitution with LAMP-2B did not rescue the autophagic flux which confirms a specific role for LAMP-2A in autophagy (Fig. S5B,C; Tables S3, S4). However, due to an absence of a control confirming a physiological role of the LAMP-2B construct we cannot definitively exclude a possible role in autophagy.

### LAMP-2 deficient cells have normal lysosome numbers and VAMP8 expression

**Fig. 3.**
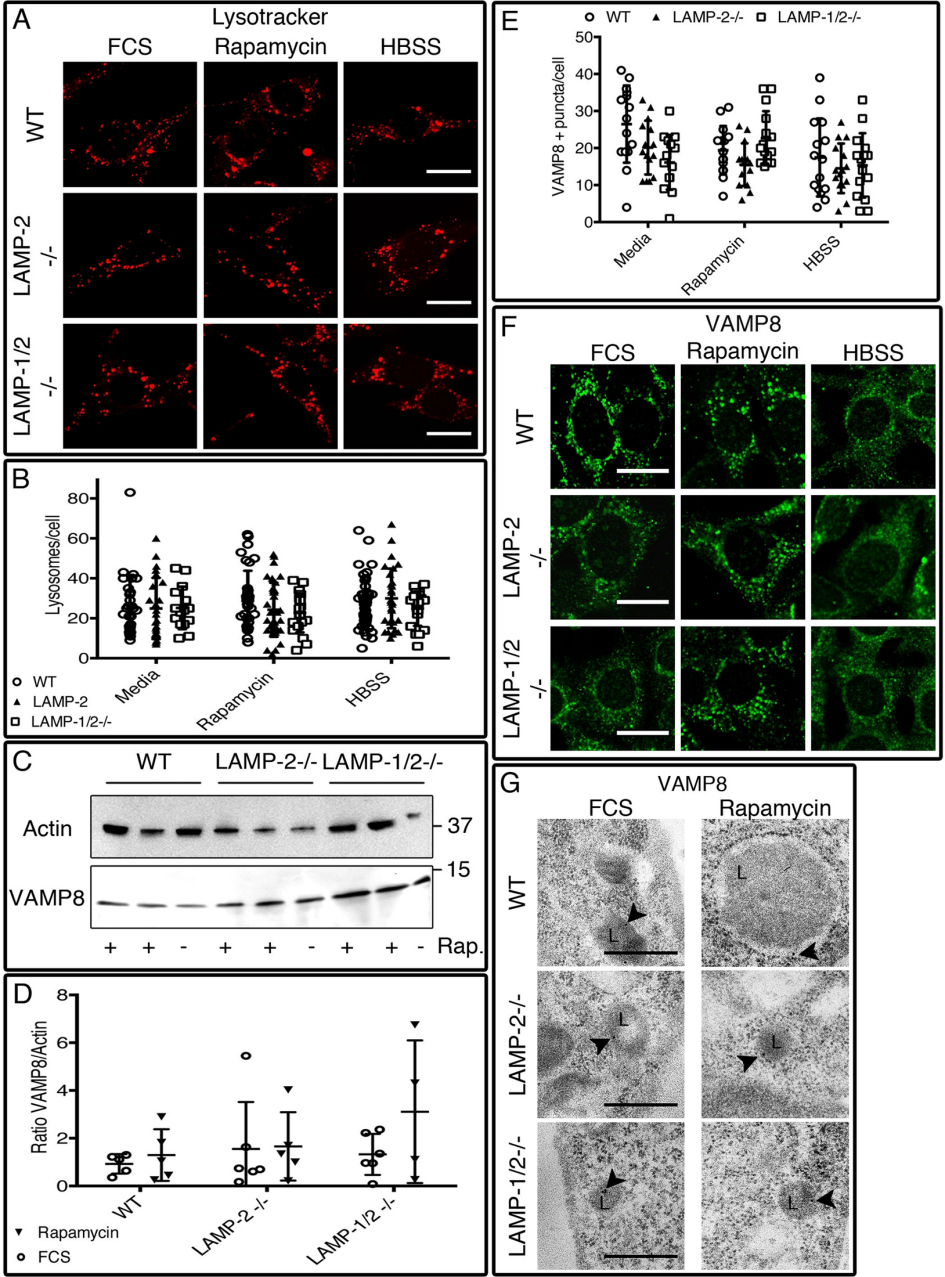
**Lysosomal parameters are not altered in the absence of LAMP-2.** (A,B) Lysosome numbers are similar in all three cell lines as is the pattern of distribution after treatment. (C,D) Expression of VAMP8 is not statistically significantly increased in LAMP-1/2-double-deficient cells. (E,F) A similar number of vesicles was observed in all cell types independent of the type of method used to induce autophagy. Scale bars=20 µm. (G) Immuno-electron microscopy confirms the localization of VAMP8 (15-nm gold particle, arrowheads) in the lysosomes of LAMP-2 sufficient and deficient cells grown in media or rapamycin. Scale bar=500nm. Data are expressed as mean±s.d. from at least 15 cells in each condition and are representative of three independent experiments. L, lysosome.

LAMP-2 is a major lysosomal membrane protein but its deficiency in the MEF cell lines, either alone or together with LAMP-1, had no effect on the number or location of lysosomes visualized with LysoTracker under resting conditions and after induction of autophagy with rapamycin or HBSS (Fig. 3A,B; Table S5). We next examined whether LAMP-2 deficiency influenced the abundance of the lysosomal SNARE VAMP8.

After rapamycin treatment, there was a non-significant increase of VAMP8 in the cell lysates from double deficient cell lines compared to wild-type and LAMP-2−/− MEFs (Fig. 3C,D). Despite this, the number of VAMP8-positive puncta was similar in all three cell lines, both before and after induction of autophagy with either rapamycin or HBSS for 6 h (Fig. 3E,F) (Table S6) or for 4 h (Fig. S6A). VAMP8 co-localized with LAMP-1, indicating it was restricted to lysosomes (Fig. S6B,C), but was not detected on LC3-positive autophagosomes (Fig. S6D,E). The specific localization of VAMP8 was confirmed by immuno-electron microscopy (Fig. 3G; Fig. S6F) where co-localization between the lysosomal marker LAMP-1 (15 nm gold particle) and VAMP8 (5 nm gold particle) was observed in both cell lines (Fig. S6G). The ratio between VAMP8 and LAMP-1, established by counting 5 and 15 nm gold particles inside LAMP-1-positive vesicle was increased non-significantly in LAMP-2 deficient cells under resting conditions and after autophagy induction. This effect was due to an increased number of VAMP8 particles as LAMP-1 remains unchanged (1.167±0377 LAMP-1 particles/ LAMP-1+ vesicles in wild-type and 1.303±1.075 LAMP-1 particles/LAMP-1+ vesicles in LAMP-2 deficient cell) (Fig. S7A). Interestingly, intact VAMP8-positive lysosome-like structures were seen inside autophagosomes of LAMP-2- and LAMP-1/2-deficient MEFs (Fig. S7B). These structures also expressed LAMP-1, confirming they were lysosomes that had been incorporated during the engulfment stage. Engulfed lysosomes were not observed in wild-type MEFs but found uniquely in singly and doubly deficient cells, either because LAMP-2-deficient lysosomes are especially susceptible to autophagy or because of the reduced autophagic flux.

The results were identical regardless of whether autophagy was induced by HBBS or rapamycin, making it highly unlikely that the LAMP-2-dependent defect in autophagy can be explained by an effect on VAMP8. Accordingly, we examined the effect of LAMP-2 deficiency on expression of STX17, VAMP8's interacting partner on autophagosomes.

### LAMP-2 deficiency prevents STX17 localization to autophagosomes

**Fig. 4.**
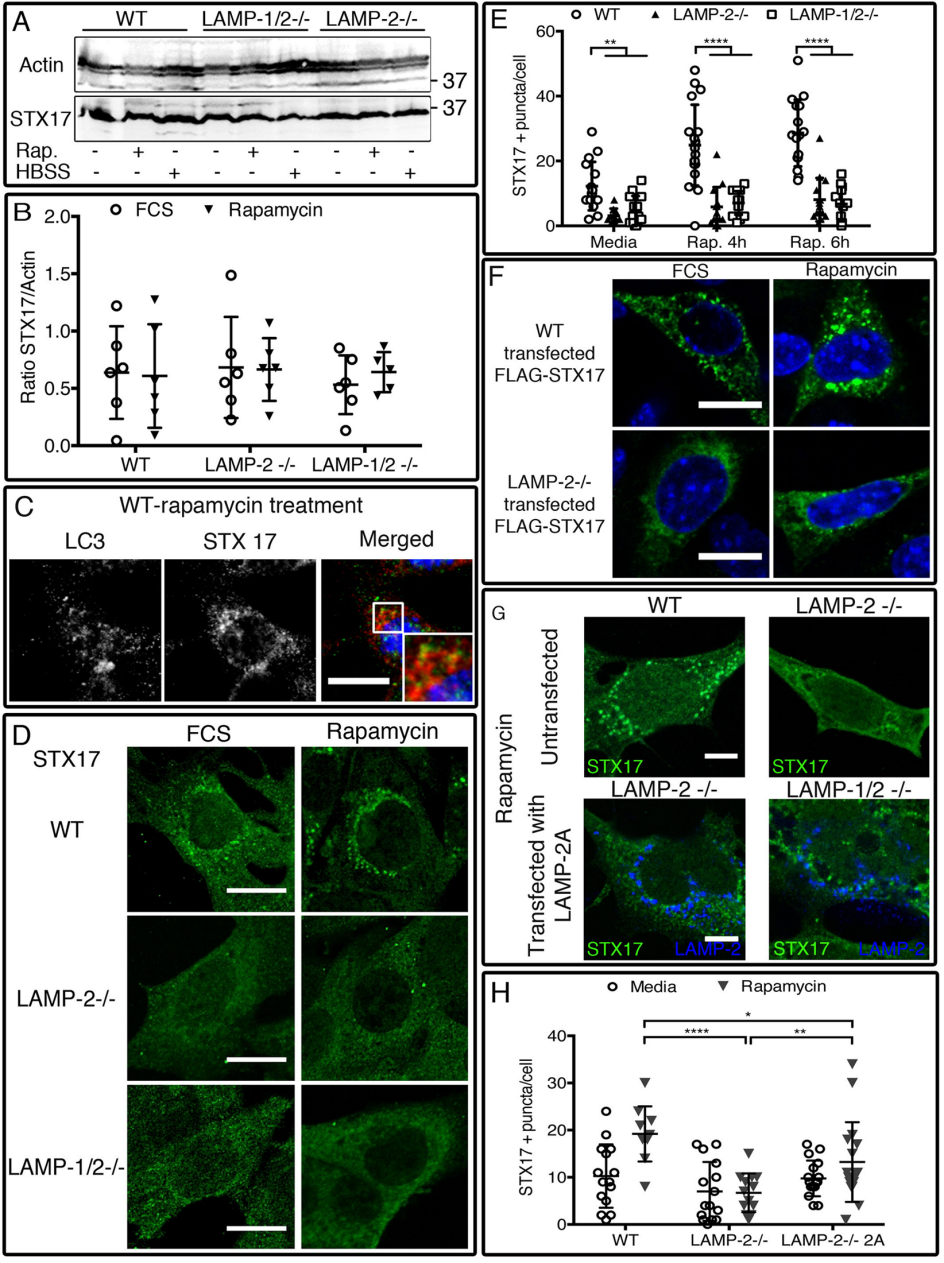
**STX17 is not found on the autophagosomes of LAMP-2 and LAMP-1/2 deficient cells.** The amount of protein in the cell lysate of LAMP-2-deficient and -sufficient cells is similar (A,B), while the localization of STX17 is different. In wild-type cells, STX17 is recruited to LC3-positive autophagosome after autophagy induction (C), but it is not observed on the autophagosomes of single- and double-deficient cells (D,E). Transfection of wild-type and LAMP-2-deficient cells with the FLAG–Stx17 construct confirms the absence of STX17 recruitment into vesicles in the absence of LAMP-2 (F). This effect could be partially restored by transfection with LAMP-2A (G,H). Scale bars=20 µm. Data are expressed as mean±s.d. of three independent experiments. *P<0.05; **P<0.01; ****P<0.0001 (Mann–Whitney test).

LAMP-2 deficiency had no effect on the abundance of STX17 in whole cell lysates from MEFs by western blot (Fig. 4A,B), and, as described by Itakura et al. (2012) and Jiang et al. (2014) it translocated to LC3+ autophagosomes in wild-type cells after induction of autophagy (Fig. 4C). Strikingly, in the absence of LAMP-2, the number of STX17-positive puncta visualized by immunocytochemistry was markedly reduced (Fig. 4D). This difference, already apparent in resting cells, was far greater after incubation with rapamycin in which STX17-positive vesicles increased in wild-type MEFs but not in the LAMP-2-deficient cell lines (Fig. 4D,E) (Table S7). We confirmed that LAMP-2-deficient cells are unable to recruit STX17 to autophagosomes by transfecting cells with FLAG-tagged STX17. Ectopic expression of human STX17 (FLAG–STX17) did not rescue STX17 translocation to LC3-positive autophagosomes (Fig. 4F) whereas reconstitution with LAMP-2A partially restored the number of STX17-positive puncta (Fig. 4G,H; Table S8). However, the increase was less than in wild-type cells, possibly due to the unstable expression levels of LAMP-2A after transient transfection, or alternatively because of the absence of the two other LAMP-2 isoforms.

**Fig. 5.**
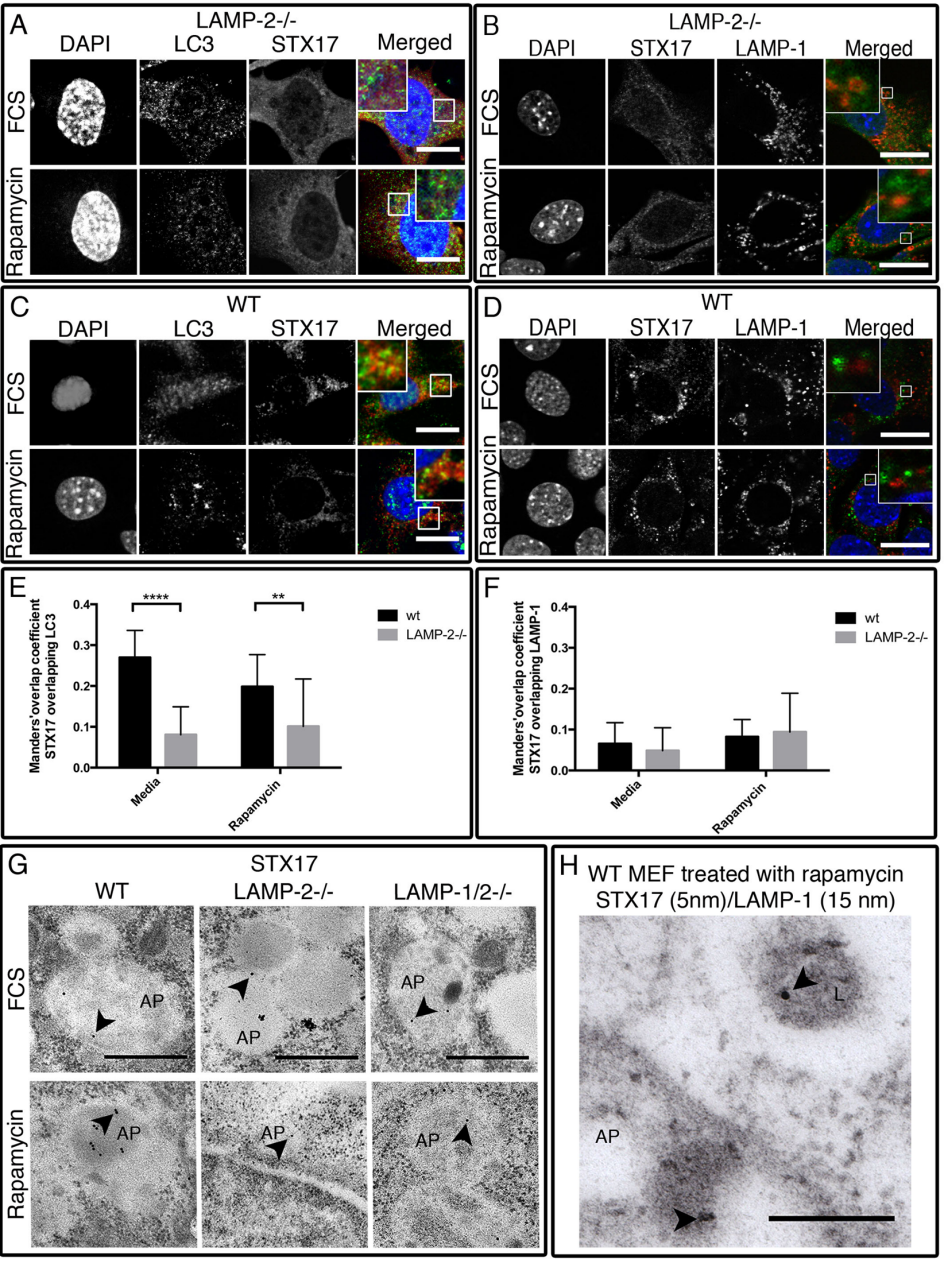
**Localization of STX17 is affected in the absence of LAMP-2.** In LAMP-2-negative fibroblasts, STX17 could not be detected on either LC3-positive autophagosomes (A) or on LAMP-1-positive vesicles (B). In wild-type MEFs, STX17 is mainly present on the autophagosomes as indicated by co-localization with LC3 (C) and occasionally on LAMP-1-positive lysosomes (D). Scale bars=20 µm. Using the JACoP plugin, a significant decrease of co-localization of STX17 with LC3 was observed in the absence of LAMP-2 (E), while the overlapping of STX17 with LAMP-1 was absent in both cell types (F). Immuno-electron microscopy confirms the autophagosomal localization of STX17 (15-nm gold particle, arrowheads) in wild-type MEFs (G). Due to the low number of STX17-positive particles definitive localization could not be established in LAMP-2-single- and LAMP-1/2-double-deficient cells. Scale bars=500 nm. (G). LAMP-1 and STX17 were observed in independent vesicles in wild-type MEFs. Scale bars=200 nm (H). Data are expressed as mean±s.d. from at least 15 cells in each condition and are representative of three independent experiments. **P<0.01; ****P<0.0001 (Mann–Whitney test). L, lysosomes; AP, autophagosome.

These results established for the first time a role of LAMP-2 in STX17 recruitment and were further confirmed by absence of STX17 co-localization on LC3-positive autophagosomes (Fig. 5A) as well as on LAMP-1-positive vesicles (Fig. 5B) in LAMP-2-deficient MEFs. In wild-type MEFs, STX17 was localized to LC3-positive vesicles (Fig. 5C) and also to rare LAMP-1-positive vesicles (Fig. 5D). Co-localization of STX17 with LC3 was significantly lower in LAMP-2-deficient cells both under resting conditions and after autophagy induction (Fig. 5E; Table S9), while overlapping of STX17 with LAMP-1 was very low in both cell lines, indicating the rare presence of STX17 on LAMP-1-positive vesicles (Fig. 5F; Table S10). The localization of STX17 was confirmed by immuno-electron microscopy, however STX17-positive autophagosomes were rare in single and double deficient fibroblasts (Fig. 5G). Rare co-localization was observed between LAMP-1 (15 nm gold particle)- and STX17 (5 nm gold particle)-positive vesicles in wild-type MEFs (Fig. 5H), whereas the ratio of STX17 particle/LAMP-1 particle was increased in LAMP-2-deficient cells treated with rapamycin (Fig. S7C). This was due to an increased number of STX17 particles, as the number of LAMP-1 particles remained unchanged (1.444±1.054 LAMP-1 particles/LAMP-1+ vesicles in wild-type and 1.221±0.518 LAMP-1 particles/ LAMP-1+ vesicles in LAMP-2-deficient cell). Moreover, the autophagosomes of LAMP-2- and LAMP-1/2-deficient MEFs also contained STX17-positive lysosome-like structures (Fig. S7D) that also expressed LAMP-1, and appeared identical to those observed with VAMP8 (Fig. S7B). Taking this observation into account, a ratio was established by quantifying the number of STX17 particles per LAMP-1-positive vesicles in lysosome-like structures and in normal vesicles. In LAMP-1-positive lysosome-like structure, the number of STX17 particles was significantly increased in LAMP-2-deficient cells after autophagy induction (Fig. S7E), while in intact vesicles the ratio was similar in each cell line independent of the treatment (Fig. S7F). Thus LAMP-2 deficiency severely reduces STX17 expression on autophagosomes and this provides a potential mechanism for their failure to fuse with lysosomes.

### LAMP-2 deficiency prevents SNAP-29 localization to autophagosomes

**Fig. 6.**
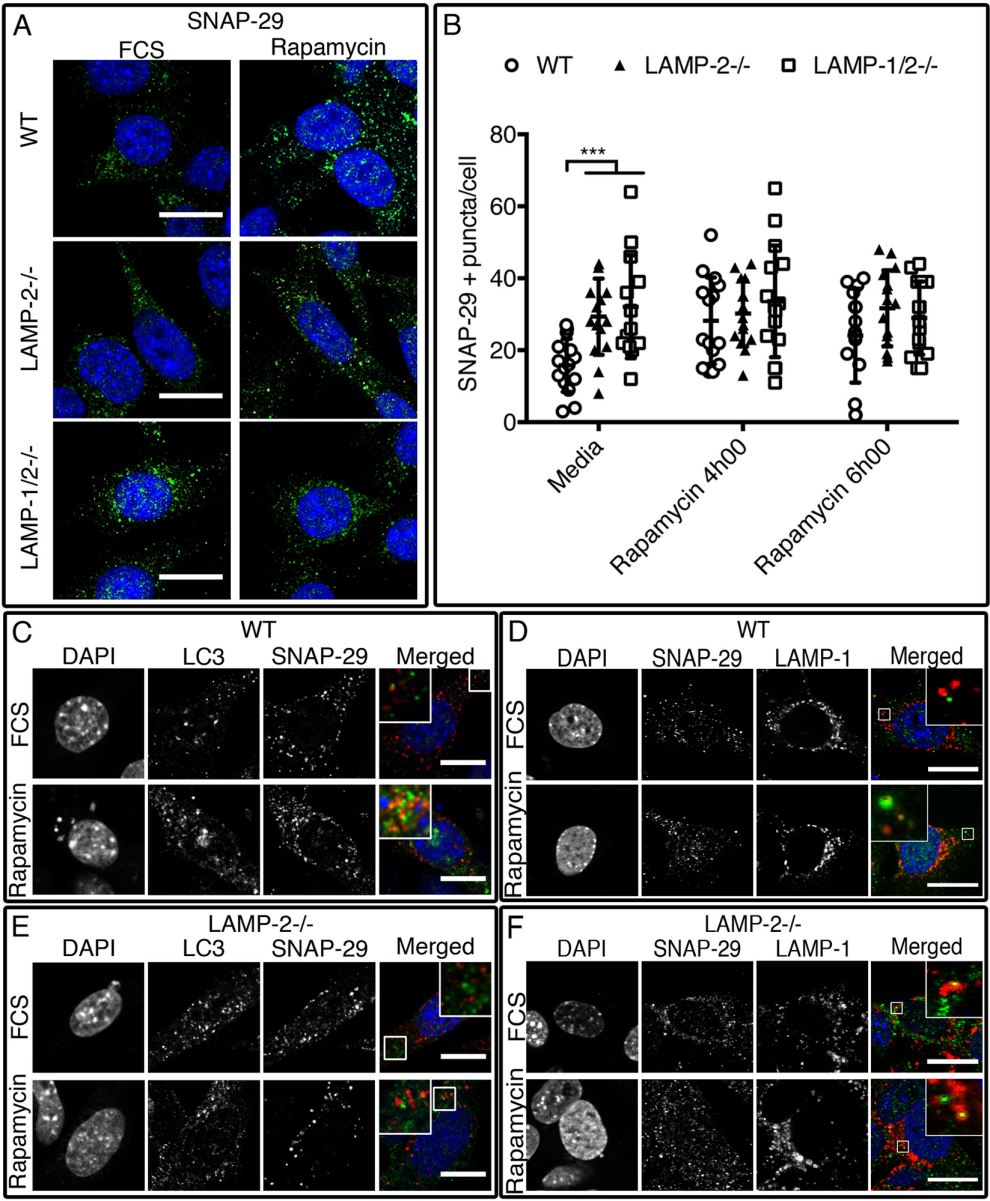
**Quantity and localization of SNAP-29**-**positive vesicles are altered in the absence of LAMP-2.** Under basal conditions more SNAP-29-positive vesicles were observed in the absence of LAMP-2 (A,B). In wild-type MEFs, SNAP-29 co-localized with EGFP–LC3-positive vacuoles (C) and occasionally with LAMP-1 (D). In LAMP-2-deficient cells, co-localization with LC3-positive vacuoles (E) was not observed while co-localization between LAMP-1 and SNAP-29 was rare (F). Scale bars=20 µm. Data are expressed as mean±s.d. of three independent experiments. ***P<0.001(Mann–Whitney test).

SNAP-29 complexes with STX17 on the surface of autophagosomes and enhances fusion by binding VAMP8 on lysosomes (Itakura et al., 2012; Steegmaier et al., 1998; Weng et al., 2007). SNAP-29-positive puncta were rare in wild-type MEFs under resting conditions and did not co-localize with LC3 (Fig. 6A,B; Table S11). After induction of autophagy, their number increased markedly and co-localization with LC3 was obvious (Fig. 6C) whereas co-localization with LAMP-1 was rare (Fig. 6D). By contrast, SNAP-29-positive puncta were significantly more common in LAMP-2-deficient MEFs under basal conditions but were unaffected by rapamycin treatment (Fig. 6A,B). SNAP-29 did not co-localize with LC3 (Fig. 6E) but occasional co-localization with LAMP-1 was observed and slightly increased after induction of autophagy (Fig. 6F) as confirmed by quantification of co-localization. By measuring co-localization we also confirmed that the overlapping of SNAP-29 with LC3 was decreased non-significantly in the absence of LAMP-2 (Mander's overlapping coefficient: 0077±0023 and 0.055±0028 in wild-type treated with FCS and rapamycin; 0.032±0.017 and 0.034±0.038 in LAMP-2-deficient cells pre- and post-rapamycin treatment). Thus, autophagosomes in LAMP-2-deficient MEFs fail to recruit SNAP-29 as well as STX17.

### VPS33A does not co-localize with LC3 in LAMP-2-deficient MEFs

The HOPS complex also binds to STX17 on autophagosomes and tethers them to Rab7 on lysosomes (Jiang et al., 2014; Lin et al., 2013) prior to fusion (Wartosch et al., 2015). Accordingly, we investigated the influence of LAMP-2 deficiency on Rab7 and the HOPS complex subunit Vacuolar protein sorting-associated protein 33A (VPS33A). The three cell lines contained similar numbers of Rab7-positive puncta (Fig. S8A,B) that also expressed LAMP-1 (Fig. S8C,D); Rab7 did not co-localize with EGFP–LC3 (Fig. S8E,F). As previously reported, Rab7-positive vacuoles were restricted to the perinuclear regions of wild-type and LAMP-2-deficient MEFs (Eskelinen et al., 2004; Jäger et al., 2004) but were more diffusely distributed in the LAMP-1/2-double-deficient cells (Fig. S8A). Thus, Rab7 retains its normal expression in lysosomes/late endosomes in LAMP-2-single-deficient MEFs.

**Fig. 7.**
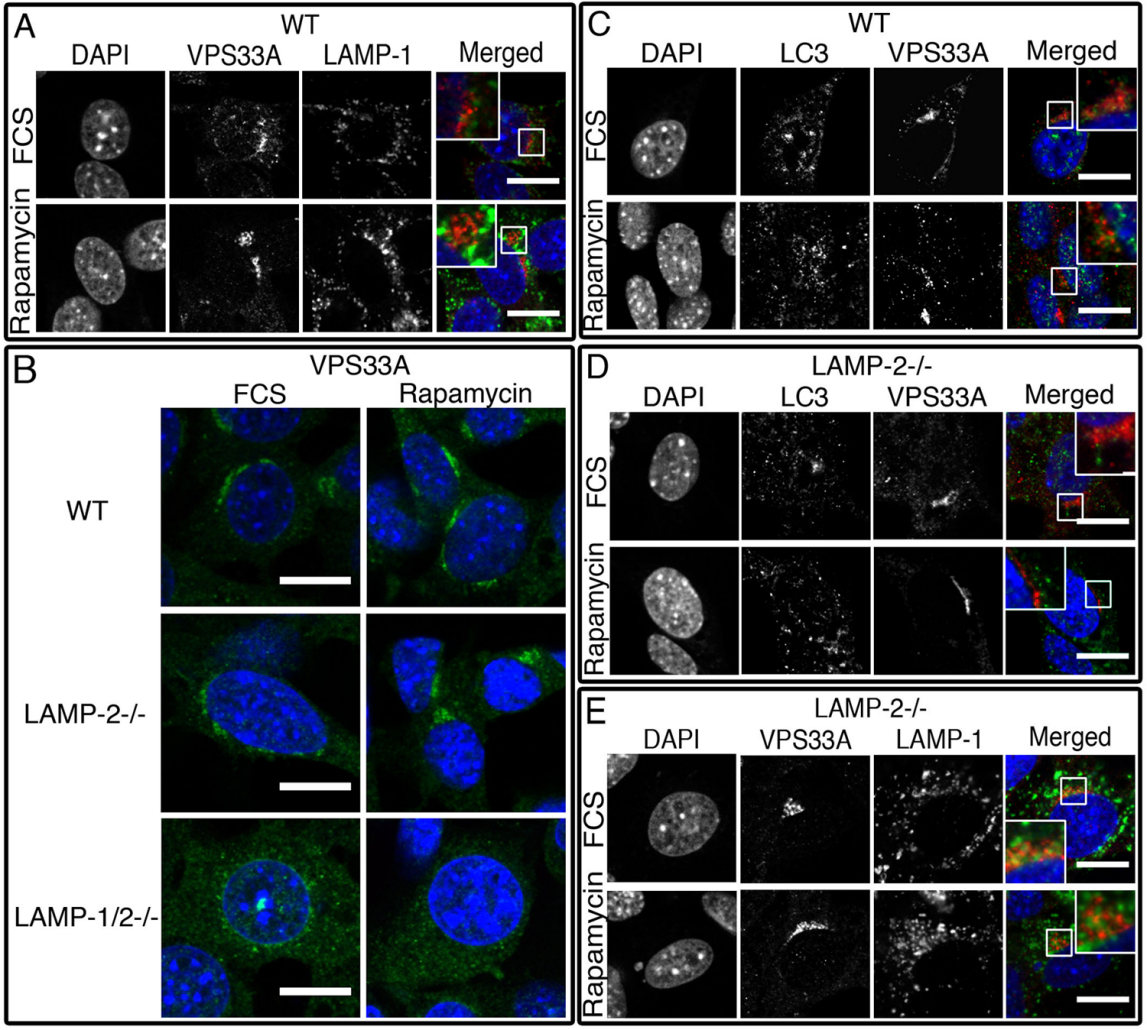
**VPS33A is affected by the absence of LAMP-2.** VPS33A partially co-localized with LAMP-1 in wild-type MEFs (A) observed as a perinuclear staining also in LAMP-2-deficient cells but absent in LAMP-1/2-double-deficient MEFs (B). After treatment with rapamycin VPS33A co-localized with EGFP–LC3 in wild-type (C) but not in LAMP-2−/− MEFs (D) where co-localization with LAMP-1 was increased (E). Scale bars=20 µm.

Under resting conditions, VPS33A partially co-localized with LAMP-1 (Fig. 7A) and had a similar perinuclear distribution in wild-type and LAMP-2-deficient MEFs (Fig. 7B). In wild-type MEFs, treatment with rapamycin induced a partial redistribution of VPS33A from LAMP-1-expressing vacuoles to LC3-positive autophagosomes (Fig. 7C) but this did not occur in LAMP-2-deficient MEFs (Fig. 7D) in which co-localization of VPS33A and LC3 was reduced, albeit not significantly (Mander's overlapping coefficient: 0.115±0.075 and 0.105±0.080 in wild-type treated with FCS and rapamycin; 0.070±0.063 and 0.082±0.042 in LAMP-2-deficient cells pre- and post-rapamycin treatment). By contrast, treatment with rapamycin increased co-localization between VPS33A and LAMP-1 in the LAMP-2-deficient cells (Fig. 7E). This provides further confirmation of the functional consequences of the lack of STX17 on autophagosomes.

## DISCUSSION

LAMP-2-deficient cells contain increased numbers of autophagosomes but it has been unclear whether this is due to increased generation or their failure to fuse with lysosomes (Eskelinen et al., 2002; González-Polo et al., 2005). Using tandem fluorescent-tagged LC3, we now show unequivocally that autophagosomes in LAMP-2-deficient mouse fibroblasts fail to fuse with lysosomes, and that the defect can be reversed by complementation with LAMP-2A, although it remains uncertain whether the other two isoforms would be similarly effective. Mechanistically, LAMP-2 deficiency reduces expression of autophagosomal SNARE STX17 to nearly undetectable levels without change in the abundance of its lysosomal partner, VAMP8, thus providing a reason for the impaired fusion (Fig. S9). These results identify the translocation of SNARE proteins to autophagosomes as a previously unsuspected function for LAMP-2 with implications not only for individuals with Danon disease who are genetically deficient of LAMP-2 but also for situations, such as aging, where LAMP-2 expression is reduced.

Cells from individuals (Danon disease) (Nishino et al., 2000) and mice (Tanaka et al., 2000) with genetic LAMP-2 deficiency have increased numbers of autophagosomes *in vivo*. LAMP-2-deficient cells *in vitro* also have increased numbers of autophagosomes but the respective contribution of enhanced or sustained formation and decreased fusion have not been clearly defined (Eskelinen et al., 2002; González-Polo et al., 2005; Massey et al., 2006). The principal difficulty has been to quantify the formation and the maturation of the autophagosomes because endogenous or GFP-tagged LC3 is immediately destroyed or quenched by lysosomal enzymes and pH after fusion and is thus rendered invisible (Kimura et al., 2007). Additional data from electron microscopy and biochemical analysis of LC3 have not proved decisive and assays for the autophagy substrate SQSTM1/p62 were not yet available (Bjørkøy et al., 2005; Eskelinen et al., 2002; Huynh et al., 2007). The development of the dual-tagged tfLC3 construct (Kimura et al., 2007) resolved these difficulties since its mRFP red component continues to fluoresce within lysosomes, enabling the determination of whether autophagosomal cargo has been degraded. This allowed us to show clearly that autophagosomal degradation was severely inhibited in the absence of LAMP-2, and to confirm this was associated with reduced autophagic flux by immunoblotting for SQSTM1/p62. The defect was equally severe in LAMP-2-single-deficient and LAMP1/2-double-deficient cells, strongly suggesting that LAMP-2 was responsible. This is supported by the fact that the defect was reversed by complementation with LAMP-2A at levels that also restored CMA, although this does not preclude contributions from other LAMP-2 isoforms.

The recent elucidation of the central components of the machinery responsible for autophagosome–lysosome fusion provided the opportunity to examine the reasons for its failure in LAMP-2-deficient cells. Fusion depends on binding of the Qa-SNARE STX17 on the autophagosomal membrane to the lysosomal R-SNARE VAMP8 (Itakura et al., 2012) in an interaction that requires the Qbc-SNARE SNAP-29 and results in the tetrameric trans-SNARE complex. The respective location of STX17 and VAMP8 to autophagosome and lysosome was first confirmed by immuno-electron microscopy. It was a surprise that deficiency of a lysosomal protein such as LAMP-2 should radically reduce STX17 expression on autophagosomes with normal, or possibly even raised VAMP8 expression on lysosomes. The absence of STX17 translocation to the autophagosomes was further supported by a reduction of STX17 co-localization with LC3 both under resting conditions and after induction of autophagy. Due to the increased numbers of autophagosomes after rapamycin treatment, co-localization of STX17 and LC3 remains unchanged under both conditions in wild-type cells. Moreover, complementation with LAMP-2A partially restores the defect, thus confirming the functional connection between LAMP-2A and STX17. However, we detected less effect of LAMP-2 deficiency on relocation of the STX17 interacting partners SNAP-29 and VPS33A to the LC3+ autophagosomes. This could either be due to technical reasons resulting from background staining with the antibodies used; or alternatively because even in the absence of their high-affinity partner STX17, SNAP-29 and VPS33A still bind to the surface of autophagosomes, albeit less efficiently. Despite a limited effect, the reduction of the Qbc SNARE SNAP-29 and HOPS complex component VPS33A on the autophagosomes supports the role of STX17 in recruiting them and identified the autophagosome as the major site of the defect. In the absence of STX17 on autophagosomes, SNAP-29 may interact and saturate its other binding partner VAMP8, explaining its lysosomal localization in LAMP-2-deficient cells. Due to saturation, induction of autophagy could not further enhance the recruitment of SNAP-29, leading to an increased number of SNAP-29-positive puncta pre- and post-rapamycin treatment. Collectively these results prove that LAMP-2 is required for normal translocation of STX17 to autophagosomes, providing the mechanism for their failure to degrade autophagosomal content.

LAMP-2 is not expressed by autophagosomes and its deficiency did not influence the whole cell content of STX17. Accordingly, LAMP-2 must be critical for translocating STX17 to autophagosomal membranes, or for its retention there. In resting cells, it has been suggested that STX17 is found freely in the cytoplasm and in the endoplasmic reticulum but translocates to mature autophagosomes, utilizing a glycine zipper-like motif in its transmembrane domain that integrates into the double membrane (Itakura et al., 2012). Membrane STX17 then recruits *O*-GlcNAcylated SNAP-29 whose availability is controlled by *O*-glycosylation transferase (OGT) (Guo et al., 2014). SNAP-29 also interacts with syntaxin 6 (Stx6) forming a SNAP-29/Stx6 SNARE complex that may regulate autophagy (Guo et al., 2014). The STX17/SNAP-29 complex is further stabilized by the incorporation of the dimeric form of Atg14 (Diao et al., 2015) which also primes its interaction with VAMP8 to promote membrane fusion (Diao et al., 2015). Finally, the HOPS complex may also stabilize the heterodimers STX17/SNAP-29 (Jiang et al., 2014). However, control of STX17's origin or availability remained unclear.

LAMP-2 is a major component of the lysosomal limiting membrane (Carlsson et al., 1988; Eskelinen et al., 2002) and has important roles in maintaining its integrity, and for transporting proteins (Eskelinen et al., 2002), nucleic acids (Fujiwara et al., 2013a) and cholesterol across it (Eskelinen et al., 2004, 2003; Wilke et al., 2012). LAMP-2 is absolutely required for CMA, which targets long-lived proteins for degradation (Cuervo and Dice, 1996; Massey et al., 2006) including inhibitors of T cell receptor signaling, which is attenuated in the absence of LAMP-2A (Valdor et al., 2014); analogous degradation of putative inhibitors of STX17 translocation would provide a potential explanation. Alternatively, abrogation of LAMP-2 dependent export of cholesterol from late endosomes and lysosomes reduces cholesterol concentrations in the endoplasmic reticulum (Schneede et al., 2011), which is known to alter recruitment of Stx6 (Reverter et al., 2014). This in turn could affect its interaction with SNAP-29 and thus its availability to interact with STX17 (Kabeya, 2000). Moreover, low levels of cholesterol in the ER and increased lysosomal cholesterol found in LAMP-2-deficient cells induce the interaction of the cholesterol sensor ORP1L with the ER protein VAP-A which prevents tethering and fusion. The ORP1L induced contact sites can modulate autophagosomes maturation and therefore potentially STX17 recruitment (Wijdeven et al., 2016). More experiments are needed to test these possibilities.

Leakage of lysosomal membranes and release of lysosomal hydrolases represent a potentially harmful situation for cells and damaged lysosomes may be specifically degraded by autophagy (lysophagy) as a protective measure (Hung et al., 2013; Maejima et al., 2013). Our data also indicate that LAMP-2 may play a role in lysophagy. The presence of LAMP-1-positive lysosomes embedded into autophagosomes of LAMP-2-deficient cells suggests either a potential role of LAMP-2 in the maintenance of the lysosomal quality and integrity or a direct contribution to autophagy-mediated lysosome turnover.

In conclusion, we have shown that LAMP-2A has a critical role in recruitment to autophagosomes of STX17 and its co-factors SNAP-29 and VPS33A and thus of fusion with lysosomes. This extends the function of LAMP-2A to include macroautophagy as well as CMA. Regardless of the mechanism, our results provide a new insight into the consequences of LAMP-2 deficiency in Danon disease. They also have implications for more common situations in which LAMP-2 concentrations are reduced sufficiently to impair its function, notably in the elderly (Cuervo and Dice, 2000; Mizushima et al., 2008; Valdor et al., 2014); and in individuals with autoantibodies to LAMP-2 and small vessel vasculitis (Kain et al., 2008; Peschel et al., 2014).

## MATERIAL AND METHODS

### Plasmids

The pEGFP–LC3, the ptfLC3 and the FLAG–Stx17 construct were acquired from Addgene (plasmid 21073, plasmid 21074 and plasmid 45911, respectively). The generation of these constructs has been previously described (Itakura et al., 2012; Kabeya, 2000; Kimura et al., 2007).

The generation of the photoswitchable reporter pKFERQ-PS-CFP2 has been described by Koga et al. (2011). The following oligonucleotides, 5′ctagcgccaccatgaaggaaactgcagcagccaagtttgagcggcagcacatggactccagcacttccgctgcg 3′ and 5′gatccgcagcggaagtgctggagtccatgtgctgccgctcaaacttggctgctgcagtttccttcatggtggcg 3′ were directly annealed and ligated into the *Nhe*I and the *Bam*HI sites of the pPS-CFP2-N vector (Evrogen, FP802, Moscow, Russia). This sequence codes for 21 amino acids of the ribonuclease A (MKETAAAKFERQHMDSSTSAA) (accession number AAB35594).

The LAMP-2A sequence (accession number NP_002285) was designed by GENEART GmbH using GeneOptimizer software. The coding sequence (0 to 1233) was based on the sequence information described by Fukuda et al. (1988). It comprises 1233 nucleotides and is bordered by 5′ggatccggagatctggggaagtctgcggggtc 3′ and 5′ctcgagggttgcagatt 3. This introduces a BamH1 site at the 5′ end and a Xho1 site at the 3′ end (underlined). These restrictions sites were used to extract the LAMP-2A fragment and clone it into the respective cloning site of pcDNA3 (Invitrogen, Carlsbad, CA, USA).

The LAMP-2B construct (accession number NP_054701) from bases –3 to 1233 was amplified from a LAMP-2B construct kindly provided by Professor Dr. Friedrich Haag, University of Hamburg, Hamburg, Germany. The PCR product generated with the primers 5′ggatccgtcatggtgtgcttccgcctcttcccg 3′ (−3 to 24) and 5′ttacagagtctgatatccagcataa 3′ (1208 to 1233) was subcloned into PCR 2.1 cloning vector using the TA cloning kit (Invitrogen, K2040-01) according to the manufacturer's instructions. The *Xho*1 and *Kpn*1 released fragment was then cloned into the respective cloning sites of pcDNA3.

### Cells culture and transfection

WT, LAMP-2−/− and LAMP-1/2−/− MEFs were a kind gift of Prof. Dr. Paul Saftig (Department of Biochemistry at the Christian-Albrechts Universität Kiel, Germany). Their generation was reported previously (Eskelinen et al., 2004). We uniquely selected cell lines immortalized by transfection with a plasmid containing the SV-40 large T antigen. Cells were cultured in Dulbecco's modified Eagles medium (DMEM) (Gibco, Waltham, MA, USA) supplemented with 10% fetal calf serum (Gibco) and 1% penicillin/streptomycin (Gibco).

MEFs were transfected using Lipofectamine 2000 (Invitrogen, 11668019), Fugene 6 (Promega, E2691, Madison, WI, USA) according to the manufacturer instructions. Transfection of the FLAG–Stx17 construct into MEFs was performed using Lipofectamine 3000 (Thermo Fisher, L3000008, Waltham, MA, USA) following manufacturer's instructions.

### Autophagy induction

Autophagy was induced by starvation or treatment with an mTOR inhibitor rapamycin (50 μM) (Calbiochem, 553210, San Diego, CA, USA) for 0, 4 and 6 h. For starvation, cells were washed three times with phosphate buffered saline (PBS) and incubated in HBSS (Invitrogen, 14025-092). Rapamycin treatment was added to fresh DMEM after three washings with PBS.

For LC3 immunostaining, rapamycin treatment was combined with chloroquine (30 µM) (Sigma, C6628, St Louis, MI, USA).

### Live cell imaging

MEFs transfected with the photoswitchable reporter pKFERQ-PS-CFP2 were cultured in µ-Slide Angiogenesis (IBIDI, 81506, Planegg, Germany). The photoconversion was carried out with a confocal laser scanning microscope (Lsm 780, Carl Zeiss, Oberkochen, Germany) by applying a 405 nm diode laser, 30 mW, 63× objective. The photoswitching time did not exceed 5 s in plane mode. The photoconversion did not affect the viability of the cells (data not shown). After treatment pictures of the cells were acquired using confocal laser scanning microscope (Lsm 780, Carl Zeiss) equipped with 63×1.4NA oil objective lens, a diode laser 405 nm and an argon laser, multiline, 458 nm, 488 nm, 514 nm, at 37C and 5% CO_2_. Pictures were analyzed with the ZEN 2010 software (Zeiss) and the quantification was done with Fiji software (Schindelin et al., 2012). Both the photoswitching and the imaging were performed in Phenol Red-free media.

### Immunostaining and confocal microscopy

MEFs were seeded on µ-Slide Angiogenesis (IBIDI, 81506) or on µ-Slide 8 well (IBIDI, 80826). After treatment with media, HBSS or rapamycin the cells were washed with PBS and fixed with freshly prepared 4% paraformaldehyde. MEFs transfected with FLAG–Stx17 were fixed and permeabilized with ice-cold methanol (10 min) followed by incubation with ice-cold acetone for 1 min. After washing with PBS, cells were permeabilized (Tables S12, S13), blocked with 10% goat serum/1% donkey serum for 30 min at room temperature and incubated with primary antibody diluted in 1% goat serum/1% donkey serum overnight at 4°C or 1 h at room temperature (Tables S12, S13). Similar diluent without primary antibody was used as negative control. After three washings with PBS, secondary antibody, diluted in goat/donkey serum 1% was added to the cells for 1 h at room temperature (Tables S12, S13). In some staining, DAPI was used to visualize the nuclei. Cells were stored in 0.4% paraformaldehyde and analyzed using a confocal laser scanning microscope (Lsm 780, Carl Zeiss) using ZEN 2010 or ZEN 2012 software. This microscope was equipped with 63×1.4NA oil objective lens, a diode laser 405 nm, an argon laser, multiline, 458 nm, 488 nm, 514 nm, a DPSS-Laser 561 nm, a HeNe-laser 594 nm and a HeNe-laser 633 nm. Images analysis and quantification were performed using Fiji software.

### Light microscopy

MEFs were cultured in four-well chamber slides. Cells were fixed in freshly prepared 4% paraformaldehyde and stained in a Benchmark Ultra Fa Ventana (Roche, Basel, Switzerland). The following protocol was used: primary antibody 32 min, hematoxylin 4 min and detection with the Universal DAB Detection Kit (Ventana, 760-500, Basel, Switzerland).

Pictures were acquired using an Axio Scope A1 microscope with a X40 objective equipped with an Axiocam ICc3 digital camera and an Axiovision analysis software.

### Immunoelectron microscopy

MEFs were seeded and cultured in Nunc cell culture Petri dishes. The fixation was performed in freshly prepared 4% paraformaldehyde and 0.1% glutaraldehyde in 0.1 M phosphate buffer for 2 h. After three washings with PBS, agarose pellets were made (1% agarose in 0.1 M phosphate buffer) and fixed in 4% paraformaldehyde and 0.1% glutaraldehyde in 0.1 M phosphate buffer for 30 min. The cells were then dehydrated in ascending alcohol sequence and embedded in acrylic resin Lowicryk K4M (Polysciences, 15923, Warrington, PA, USA). After blocking with 1% chicken egg albumin, ultrathin sections were treated with rat anti-mouse LAMP-1 clone 1DB4 (Developmental Studies Hybridoma Bank, Iowa City, IA, USA) and/or rabbit anti-VAMP8 (HPA006882, Sigma, 1:10) or rabbit anti-STX17(HPA001204, Sigma, 1:5) for 2 h. After three washings in 0.1% chicken egg albumin in 0.1 M Tris, cells were incubated for 1 h00 with goat anti-rabbit IgG conjugated to colloidal gold (15 nM) (BBInternational, EM.GAR15, Cardiff, UK) and doubly stained with 2% uranyl acetate (Ted Pella, Inc., 19481, Redding, CA, USA) and lead citrate. For double staining, goat anti-rabbit IgG conjugated to colloidal gold particle of 5 nM and goat anti-rat IgG conjugated to colloidal gold particles of 15nM were used. Sections were examined with a JEOL JEM-1200EX transmissions electron microscope.

Quantitative immuno-electron microscopy was performed by counting the number of colloidal gold particles (5 and 15 nM). All quantifications were done by an observer blinded to experimental conditions.

### Western blot

Expression of LAMP-1, LAMP-2, actin, VAMP8 and STX17 was analyzed in MEF homogenates. Cells were collected in PBS after trypsinization, sedimented by spinning, resuspended in lysis buffer (125 mM Tris-HCl pH 6.8/4% SDS/20% glycerol/200 mM DTT) and subjected to sonication. After heating for 5 min at 95°C, protein fraction of MEFs was separated by a 10% SDS-PAGE (LAMP-1 and LAMP-2) or by a 10-20% Ready Gel Tris-HCl (Bio-Rad, 161-1160, Hercules, CA, USA) and transferred onto a polyvinylidene difluoride membrane. After blocking for 1 h at room temperature, the blot was incubated overnight at 4°C with primary antibody. Membranes were washed three times for 5 min with TBS-Tween (0.05%) and incubated with alkaline phosphatase coupled antibody for 1 h at room temperature. After incubation with the DuoLux chemiluminescence substrate (Vector Laboratories, SK 6604, Burlingame, CA, USA) the signal was analyzed using the Lumi-Imager F1. Image processing and western blot quantification were done by using Fiji software. Results represent the quantification of at least four independent experiments and are expressed as relative to the level of the housekeeper protein actin.

### Antibodies

The following primary antibodies were used in this study: rat anti-mouse LAMP-1 (clone 1DB4, Developmental Studies Hybridoma Bank, Iowa City, IA, USA; Huynh et al., 2007); rat anti-mouse LAMP-2 (clone ABL-93, Developmental Studies Hybridoma Bank; Eskelinen et al., 2002; Schneede et al., 2011); rabbit anti-SNAP-29 (Sigma, HPA031823; specificity extensively characterized by the Human Protein Atlas); rabbit anti-VAMP8 (Sigma, HPA006882; characterized by the Human Protein Atlas), rabbit anti-syntaxin17 (Sigma, HPA001204; characterized by the Human Protein Atlas; Diao et al., 2015; Jiang et al., 2014; Muppirala et al., 2012); rabbit anti-VPS33A [C1C3] (GeneTex, GTX 119416, Taiwan; van der Kant et al., 2013), goat anti-MAP LC3α/β (F-14, sc-16756, Santa Cruz, Dallas, TX, USA; immunofluorescence; Gohla et al., 2007; Tannous et al., 2008a,b); mouse anti-SV-40 monoclonal antibody (Cell Marque, 351M-17, Rocklin, CA, USA); human LAMP-2 (clone H4B4, Developmental Studies Hybridoma Bank; Peschel et al., 2014); rabbit anti-actin (Sigma, A2066; Miyauchi et al., 2010); rabbit anti-p62 (Cell Signaling, 5114, Danvers, MA, USA; Fang et al., 2013); rabbit anti-LC3B (Cell Signaling, 2775; western blot; Zhou et al., 2012); rabbit anti-Rab7 (Cell Signaling, 9367; immunofluorescence; Jean et al., 2015); rabbit anti-FLAG (Sigma, F7425; Carmona-Mora et al., 2015; Goodchild et al., 2015).

For immunofluorescence we used the following secondary antibodies: Alexa Fluor 488 goat anti-rat IgG (Invitrogen, A-11006), Alexa Fluor 546 goat anti-rat IgG (Invitrogen, A-11081), Alexa Fluor 488 goat anti-rabbit IgG (Invitrogen, A-11008), Alexa Fluor 647 goat anti-rabbit IgG (Invitrogen, A-21244), Alexa Fluor 405 goat anti-mouse IgG (Invitrogen, A-31553), Alexa Fluor 546 goat anti-mouse IgG (Invitrogen, A-11003), Alexa Fluor 594 donkey anti-rabbit IgG (Thermo Fisher, A-21207), Alexa Fluor 488 donkey anti-goat IgG (Thermo Fisher, A-11055). Anti-rat IgG-AP conjugate (Promega, S3831) and anti-rabbit IgG-AP conjugate (Promega, S3738) were used for western blot.

### Quantification of lysosomes

Lysosomes were labeled with LysoTracker (LysoTracker Red DND-99, Invitrogen, L7528). Briefly, the cells, cultured in a µ-slide angiogenesis (IBIDI, 81506) were treated with rapamycin or HBSS for 0, 4 and 6 h. Then 50 nM of LysoTracker was added to the media for 30 min at 37°C and the cells were fixed in freshly prepared 4% paraformaldehyde in PBS. The number of lysosomes was evaluated using a particle counting plugin in Fiji software.

### Image analysis

Positively transfected cells were identified by eye. For each quantification, a region of interest of one cell was delineated including the cytoplasm and excluding the nucleus. All quantifications were performed by an observer blinded to experimental conditions.

Quantification of mRFP/EGFP colocalization was performed by using the plugin ‘Coloc 2'on the Fiji software.

The number of EGFP-positive and mRFP-positive puncta observed after transfection with ptfLC3 were quantified using the function 3D Object Counter v2.0 on the Fiji software. Similar size filter was applied through the whole experiment while the threshold was determined individually by a blinded experimenter; the transient transfection leading to various level of expression.

The number of EGFP-positive puncta in fibroblast transfected with the photoswitchable reporter pKFERQ-PS-CFP2 was counted by eye.

Quantification of the positive vesicles stained with a LysoTracker or by immunofluorescence was operated by a counting plugin with filters for threshold and size, adapted for each experiment. Similar filters were applied for each condition inside each experiment.

Quantitative co-localization analysis evaluating the proportion of SNAP-29/STX17/VPS33A colocalizing with LAMP-1/LC3 was performed over the entire fluorescence images using the Fidji's plugin ‘JACoP’ (Just Another Colocalization Plugin; Bolte and Cordelieres, 2006).

### Statistical analysis

The statistical significance of the CMA experiment was evaluated by a one-way ANOVA followed by a Student's *t*-test. The other experiments were analyzed using a non-parametrical Kruskal–Wallis test followed by a Mann–Whitney test when appropriate. Values are presented as mean±s.d. All statistical calculations were performed using GraphPad Prism software.
